# Supplementary material for: Characteristics, Source Contributions, and Source-Specific Health Risks of PM2.5-Bound Polycyclic Aromatic Hydrocarbons for Senior Citizens during the Heating Season in Tianjin, China
Source: Int J Environ Res Public Health. 2022 Apr 7;19(8):4440. doi: 10.3390/ijerph19084440 (PMC9030979; doi:10.3390/ijerph19084440)
Supplement: Supplementary file 1 [file ijerph-19-04440-s001.zip › ijerph-1644907-supplementary.pdf]

Supporting Information (SI) of

Characteristics, Source Contributions, and Source-Specific Health Risks of $PM_{2.5}$-Bound Polycyclic Aromatic Hydrocarbons for Senior Citizens during the Heating Season in Tianjin, China

Nan Zhang [1,†], Chunmei Geng [1,†], Jia Xu [1], Liwen Zhang [2], Penghui Li [3], Jinbao Han [4], Shuang Gao [5], Xinhua Wang [1], Wen Yang [1], Zhipeng Bai [1], Wenge Zhang [6,*] and Bin Han [1,*]

[1] State Key Laboratory of Environmental Criteria and Risk Assessment, Chinese Research Academy of Environmental Sciences, Beijing 100012, China; zhangnan01@craes.org.cn (N.Z.); gengcm@craes.org.cn (C.G.); carol3233@126.com (J.X.); wangxh@craes.org.cn (X.W.); yangwen@craes.org.cn (W.Y.); baizp@craes.org.cn (Z.B.)

[2] Department of Occupational and Environmental Health, School of Public Health, Tianjin Medical University, Tianjin 300070, China; zhangliwen@tmu.edu.cn

[3] School of Environmental Science and Safety Engineering, Tianjin University of Technology, Tianjin 300384, China; lipenghui406@163.com

[4] School of Quality and Technical Supervision, Hebei University, Baoding 071002, China; jinbaobaohan@163.com

[5] School of Geographic and Environmental Sciences, Tianjin Normal University, Tianjin 300387, China; shuang1gao@163.com

[6] Particle Laboratory, Center for Environmental Metrology, National Institute of Metrology, Beijing 100022, China

* Correspondence: zhangwg@nim.ac.cn (W.Z.); hanbin@craes.org.cn (B.H.)

† These authors contributed equally to this work.

Table S1 General information of participants

Table S1 gave the general information of 101 participants

Table S1. General information of participants (N=101)

Characteristic		N	%	Time of daily activity (h)			
				Mean	SD	Min	Max
Sex	Male	41	40.6	/	/	/	/
	Female	60	59.4	/	/	/	/
Smoking	Yes	15	14.9	0.6	0.4	0.1	1.7
	No	86	85.1	/	/	/	/
ETS exposed	Yes	29	28.7	0.6	0.4	0.1	1.7
	No	72	71.3	/	/	/	/
Cooking	Yes	72	71.3	1.5	0.9	2	4
	No	29	28.7	/	/	/	/

ETS: environmental tobacco smoking; SD: standard deviation

Text S1 PAHs analysis procedure and quality assurance and quality control

PAHs were analyzed using gas chromatography coupled with mass spectrometry method (GC/MS, trace 2000 GC-MS, USA). The filter was extracted with dichloromethane by the ultrasonicator. The extract was firstly concentrated to approximately 5 ml by the rotary evaporator, and then condensed to 1 ml under the gentle flow of pure nitrogen. The condensed extract was injected to the gas chromatography coupled with mass spectrometer, and the PAHs were separated and identified based on the retention time.

Two types of standard reference materials (Supelco #48743 and Supelco #36962, Environmental Protection Agency, USA) were used in the PAHs analysis, and the information of internal standards, method detection limit, recovery and relative standard deviation was listed in Table S2.

Table S2. Summary of the PAH analysis and Quality Control/Quality Assurance.

PAH	Internal Standard	Method Detection Limit (ng/ml)	Recovery (%)	Relative Standard Deviation (%)
Flu	D_{10}-Acenaphthene	6	86.6	7.7
Phe	D_{10}-Phenanthrene	8	89.6	3.8
Pyr	D_{12}-Chrysene	6	84.5	5.6
BaA	D_{12}-Chrysene	10	81.2	6.8
BbF	D_{10}-Acenaphthene	10	84.9	9.2
Chr	D_{12}-Chrysene	10	84.9	7.1
BeP	D_{12}-Perylene	12	88.8	4.3
BaP	D_{12}-Perylene	12	92.7	6.8
IND	D_{10}-Acenaphthene	10	81.2	12.7
BghiP	D_{10}-Acenaphthene	10	82.5	14.5
Fl	D_{10}-Phenanthrene	7	88.0	9.9
BkF	D_{12}-Perylene	10	87.08	9.63
Ace	D_{10}-Acenaphthene	7	89.72	8.65
DahA	D_{10}-Acenaphthene	10	82.11	10.61

Figure S1 Twenty-four hours air parcel backward trajectories (every 2 h in each day) during sampling period. The end point is at 500 m AGL at the selected residential communities (N: 39.10°, E: 117.16°). The percent of trajectories belonging to a particular cluster is shown next to the line.

Figure S1 showed the air parcel backward trajectories during the sampling period. During winter, there was no trajectories coming from the sea and the personal $PM_{2.5}$ samples in this paragraph were hardly influenced by sea breeze.

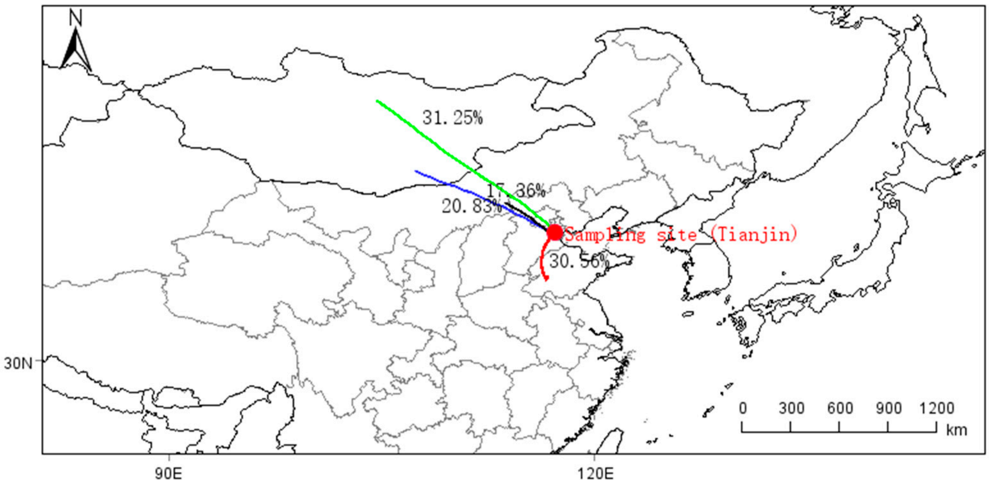

Figure S1. Twenty-four hours air parcel backward trajectories (every 2 h in each day) during sampling period. The end point is at 500 m AGL at the selected residential communities (N: 39.10°, E: 117.16°). The percent of trajectories belonging to a particular cluster is shown next to the line.
